# Supplementary material for: Dysfunction of the episodic memory network in the Alzheimer’s disease cascade
Source: Nat Commun. 2026 Apr 17;17:3578. doi: 10.1038/s41467-026-71831-z (PMC13090369; doi:10.1038/s41467-026-71831-z)
Supplement: Supplementary file 1 — Supplementary Information [file 41467_2026_71831_MOESM1_ESM.pdf]

Supplementary material to

"Dysfunction of the episodic memory network in the Alzheimer's disease cascade"

Lattmann et al.

Supplementary Table 1 | Baseline characteristics of the final analysis sample (n = 493)

	Missing	CN (n = 165)	SCD (n = 214)	MCI (n = 82)	DAT (n = 32)	Group test P-value	Pairwise comparisons
Age (y)	-	69.17 (5.24)	70.51 (6.05)	72.72 (5.65)	73.86 (6.26)	< 0.001	CN vs. SCD, MCI, DAT<br>SCD vs. MCI, DAT
Sex (% female)	-	105 (63.64)	104 (48.60)	43 (52.44)	18 (56.25)	0.041	CN vs. SCD
Education (y)	-	14.61 (2.68)	15.28 (2.93)	13.90 (3.02)	13.09 (2.86)	< 0.001	CN vs. DAT<br>SCD vs. MCI, DAT
APOE ε4 carrier (%)	5 (1.01)	38 (23.60)	70 (32.86)	41 (50.00)	21 (65.63)	< 0.001	CN vs. MCI, DAT<br>SCD vs. MCI, DAT
CSF-Aβ42/40 ratio	275 (55.78)	0.10 (0.02)	0.09 (0.03)	0.07 (0.03)	0.05 (0.02)	< 0.001†	CN vs. MCI, DAT<br>SCD vs. MCI, DAT<br>MCI vs. DAT
CSF-pTau$_{181}$ (pg/ml)	275 (55.78)	45.86 (13.51)	51.68 (20.50)	68.02 (34.00)	99.06 (42.55)	< 0.001†	CN vs. MCI, DAT<br>SCD vs. MCI, DAT<br>MCI vs. DAT
Follow-up time (years)							
fMRI data	0	1.95 (1.30)	1.73 (1.16)	1.17 (1.04)	.56 (.84)	< 0.001†	CN vs. SCD, MCI, DAT<br>SCD vs. MCI, DAT<br>MCI vs. DAT

The groups were significantly different in terms of age ($F_{3,489}$ = 10.59, $p = 9.312\times10^{-7}$), sex (χ^2_3 = 8.29, $p = 4.038\times10^{-2}$), years of education ($F_{3,489}$ = 8.45; $p = 1.750\times10^{-5}$) and APOE ε4 carriership (χ^2_3 = 31.26, $p = 7.494\times10^{-7}$). Additionally, CSF-Aβ42/40 ratios ($F_{3,76.297}$ = 52.17, p 1.911×10^{-18}) and CSF-pTau$_{181}$ levels ($F_{3,62.296}$ = 14.60, $p = 2.624\times10^{-7}$) were different across the groups. Finally, follow-up time was significantly different across the groups ($F_{3,132.41}$ = 25.189, $p = 5.870\times10^{-13}$). CN = healthy controls, SCD = subjective cognitive decline, MCI = mild cognitive impairment, DAT = mild dementia of the Alzheimer's disease type. †F-test calculated using the Welch method. Pairwise comparisons denote significant post-hoc differences after Bonferroni-Holm correction using two-sided t-tests

Supplementary Table 2 | Baseline characteristics of the model training sample (n = 208)

	Missing	CN (n = 80)	SCD (n = 57)	MCI (n = 44)	DAT (n = 27)	Group test P-value	Pairwise comparisons
Age (y)	-	69.22 (4.84)	72.66 (5.16)	73.84 (5.38)	75.11 (5.57)	< 0.001	CN vs. SCD, MCI, DAT
Sex (% female)	-	41 (51.25)	21 (38.84)	21 (47.73)	16 (59.26)	0.208	-
Education (y)	-	14.36 (2.67)	15.25 (3.02)	13.93 (2.90)	12.74 (2.57)	< 0.001	SCD vs. AD
APOE ε4 carrier (%)	1 (0.5)	19 (23.75)	38 (67.86)	30 (68.18)	17 (62.06)	< 0.001	CN vs. SCD, MCI, DAT
CSF-Aβ42/40 ratio	-	0.10 (0.02)	0.06 (0.01)	0.05 (0.01)	0.05 (0.01)	< 0.001†	CN vs. SCD,MCI, DAT<br>SCD vs. MCI, DAT
CSF-pTau$_{181}$ (pg/ml)	-	46.83 (14.39)	72.79 (30.06)	84.04 (29.18)	89.61 (28.14)	< 0.001†	CN vs. SCD,MCI, DAT<br>SCD vs. MCI, DAT
Follow-up time (years)							
CSF	421 (56)	1.56 (1.68)	1.24 (1.74)	1.00 (1.74)	.32 (.97)	< 0.001†	CN vs. DAT
MTL Volumes	205 (27)	2.25 (1.16)	1.71 (1.28)	1.35 (1.39)	.63 (1.14)	< 0.001†	CN vs. MCI, DAT<br>SCD vs. DAT
PACC5	89 (12)	3.54 (2.13)	2.50 (1.99)	1.44 (1.87)	.44 (.96)	< 0.001†	CN vs. SCD, MCI, DAT<br>SCD vs. MCI, DAT<br>MCI vs. DAT
ADAS-COG-13	33 (4)	3.53 (2.32)	2.57 (2.03)	2.13 (2.32)	1.16 (1.74)	< 0.001	CN vs. SCD, MCI, DAT<br>SCD vs. AD

The groups were significantly different in terms of age ($F_{3,204} = 13.27$, $p = 6.002\times10^{-8}$), years of education ($F_{3,204} = 5.14$, $p = 1.903\times10^{-3}$), APOE ε4 carriership ($\chi^2_3 = 36.82$, $p = 5.023\times10^{-8}$), CSF-Aβ42/40 ratio ($F_{3,96.875} = 100.71$, $p = 1.152\times10^{-29}$), and CSF-pTau$_{181}$ ($F_{3,73.993} = 42.36$, $p = 4.809\times10^{-16}$). Sex distributions were not different across the groups ($\chi^2_3 = 4.54$, p = .208). For the longitudinal data, CSF ($F_{3,97.152} = 7.6592$, $p = 1.199\times10^{-4}$), volume ($F_{3,87.992} = 13.773$, $p = 1.924\times10^{-7}$), PACC5 ($F_{3,102.78} = 38.858$, $p = 7.209\times10^{-17}$), and ADAS-COG-13 ($F_{3,204} = 10.25$, $p = 2.575\times10^{-6}$) follow-up times were significantly different across the groups. Pairwise comparisons denote significant post-hoc differences after Bonferroni-Holm correction using two-sided t-tests. CN = healthy controls, SCD = subjective cognitive decline, MCI = mild cognitive impairment, DAT = mild dementia of the Alzheimer's disease type. †F-test calculated using the Welch method.

Supplementary Table 3 | *Post-hoc* comparisons for the association between disease stage and diagnostic groups

Group Comparison	Mean Difference	SE	P_{uncorr}	P_{corr}
SCD > CN	1.53	0.359	1.22×10^{-4}	1.22×10^{-4}
MCI > CN	5.85	0.694	5.37×10^{-25}	2.15×10^{-24}
DAT > CN	12.91	0.755	2.72×10^{-50}	1.63×10^{-49}
MCI > SCD	4.32	0.686	4.03×10^{-16}	8.06×10^{-16}
DAT > SCD	11.38	0.747	1.57×10^{-42}	7.83×10^{-42}
DAT > MCI	7.06	0.954	1.43×10^{-16}	4.29×10^{-16}

Pairwise comparisons from the ANCOVA assessing the association between diagnostic groups and disease stage corrected for age, sex, education and scanning site. Statistical significance was evaluated using one-sided independent-sample t-tests. P_{uncorr} = raw p-values. P_{corr} = corrected p-values using Bonferroni-Holm. CN = healthy controls, SCD = subjective cognitive decline, MCI = mild cognitive impairment, DAT = mild dementia of the Alzheimer's disease type.

Supplementary Table 4 | *Post-hoc* comparisons for the association between disease stage and AT staging groups

Group Comparison	Mean Difference	SE	P_{uncorr}	P_{corr}
A+T- > A-T-	3.27	0.733	1.56×10^{-6}	1.56×10^{-6}
A+T+ > A-T-	9.1	0.716	1.21×10^{-29}	3.64×10^{-29}
A+T+ > A+T-	5.83	0.916	2.34×10^{-11}	4.68×10^{-11}

Pairwise comparisons from the ANCOVA assessing the association between AT staging groups and disease stage adjusting for age, sex, education, and scanning site. Statistical significance was evaluated using one-sided independent-samples t-tests. P_{uncorr} = raw p-values. P_{corr} = corrected p-values using Bonferroni-Holm. A-T- = amyloid and tau negative, A+T- = amyloid positive and tau negative, A+T+ = amyloid and tau positive.

Supplementary Table 5 | Parallel Mediation analyses – Disease-related deactivation

Estimator	ML
Optimization method	NLMINB
Number of model parameters	15
Number of observations	407

Model Test User Model:

Test statistic	12.423
Degrees of freedom	5
P-value (Chi-square)	0.029

Model Test Baseline Model:

Test statistic	282.879
Degrees of freedom	15
P-value	0.000

User Model versus Baseline Model:

Comparative Fit Index (CFI)	0.972
Tucker-Lewis Index (TLI)	0.917

Loglikelihood and Information Criteria:

Loglikelihood user model (H0)	-2092.047
Loglikelihood unrestricted model (H1)	-2085.836
Akaike (AIC)	4214.095
Bayesian (BIC)	4274.227
Sample-size adjusted Bayesian (SABIC)	4226.630

Root Mean Square Error of Approximation:

RMSEA	0.060
90 Percent confidence interval - lower	0.018
90 Percent confidence interval - upper	0.104
P-value H_0: RMSEA <= 0.050	0.289
P-value H_0: RMSEA >= 0.080	0.257

Standardized Root Mean Square Residual:

SRMR	0.045

Parameter Estimates:

Standard errors	Bootstrap
Number of requested bootstrap draws	5000
Number of successful bootstrap draws	5000

Regressions:

	Est.	Std.Err	z-value	P(>|z|)	Std.lv	Std.all
Cognition ~						
Deactivation(c_pr)	-0.223	0.046	-4.800	0.000	-0.223	-0.226
HC_vol ~						
Deactivation. (a1)	0.243	0.059	4.112	0.000	0.243.	0.237
EC_vol ~						
Deactivation (a2)	0.201	0.052	3.866	0.000	0.201	0.196
WMH ~						
Deactivation (a3)	0.105	0.044	2.390	0.017	0.105	0.109
EffCnn ~						
Deactivation (a4)	0.045	0.015	3.095	0.002	0.045	0.204
Cognition ~						
HC_vol (b1)	-0.153	0.054	-2.836	0.005	-0.153	-0.158
EC_vol (b2)	-0.107	0.052	-2.062	0.039	-0.107	-0.111
WMH (b3)	-0.086	0.046	-1.878	0.060	-0.086	-0.085
EffCnn (b4)	-0.718	0.226	-3.177	0.001	-0.718	-0.161

Covariances:

	Est.	Std.Err	z-value	P(>|z|)	Std.lv	Std.all
.HC_vol ~~						
.EC_vol	0.476	0.055	8.633	0.000	0.476	0.501

Variances:

	Est.	Std.Err	z-value	P(>|z|)	Std.lv	Std.all
.Cognition	0.750	0.054	14.004	0.000	0.750	0.809
.HC_vol	0.941	0.072	13.053	0.000	0.941	0.944
.EC_vol	0.959	0.072	13.258	0.000	0.959	0.961
.WMH	0.884	0.058	15.211	0.000	0.884	0.988
.EffCnn	0.045	0.003	13.873	0.000	0.045	0.959

Defined Parameters:

	Est.	Std.Err	z-value	P(>|z|)	Std.lv	Std.all
indirect_HC	-0.037	0.017	-2.211	0.027	-0.037	-0.038
indirect_EC	-0.021	0.012	-1.814	0.070	-0.021	-0.022
indirect_WMH	-0.009	0.006	-1.414	0.157	-0.009	-0.009
indirect_EffCnn	-0.032	0.014	-2.354	0.019	-0.032	-0.033
total_indirect	-0.100	0.025	-3.964	0.000	-0.100	-0.101
direct	-0.223	0.046	-4.799	0.000	-0.223	-0.226
total	-0.323	0.052	-6.225	0.000	-0.323	-0.327

Output for the parallel mediation analysis using data from n = 407 individuals with complete cognitive, connectivity, WMH, volume and fMRI data at baseline for disease-related deactivation. Parameters were tested using the two-sided Wald z-statistic. Please note that p-values are uncorrected. Abbreviations: EC_vol – Entorhinal Cortex Volume; EffCnn – Effective Connectivity; HC_vol – Hippocampal Volume; indirect_HC – Indirect path via Hippocampal Volume; indirect_EC – Indirect Path via Entorhinal Cortex Volume; indirect_WMH – Indirect Path via White Matter Hyperintensities; indirect_WMH – Indirect Path via Effective Connectivity; WMH – White Matter Hyperintensities

Supplementary Table 6 | Parallel Mediation analyses – Disease-related activation

Estimator	ML
Optimization method	NLMINB
Number of model parameters	15
Number of observations	407

Model Test User Model:

Test statistic	15.902
Degrees of freedom	5
P-value (Chi-square)	0.007

Model Test Baseline Model:

Test statistic	260.580
Degrees of freedom	15
P-value	0.000

User Model versus Baseline Model:

Comparative Fit Index (CFI)	0.956
Tucker-Lewis Index (TLI)	0.867

Loglikelihood and Information Criteria:

Loglikelihood user model (H0)	-2104.936
Loglikelihood unrestricted model (H1)	-2096.985
Akaike (AIC)	4239.873
Bayesian (BIC)	4300.005
Sample-size adjusted Bayesian (SABIC)	4252.408

Root Mean Square Error of Approximation:

RMSEA	0.073
90 Percent confidence interval - lower	0.035
90 Percent confidence interval - upper	0.115
P-value H_0: RMSEA <= 0.050	0.143
P-value H_0: RMSEA >= 0.080	0.439

Standardized Root Mean Square Residual:

SRMR	0.053

Parameter Estimates:

Standard errors	Bootstrap
Number of requested bootstrap draws	5000
Number of successful bootstrap draws	5000

Regressions:

	Est.	Std.Err	z-value	P(>|z|)	Std.lv	Std.all
Cognition ~						
Activation (c_pr)	0.214	0.052	4.117	0.000	0.214	0.216
HC_vol ~						
Activation (a1)	-0.172	0.056	-3.094	0.002	-0.172	-0.167
EC_vol ~						
Activation (a2)	-0.167	0.058	-2.883	0.004	-0.167	-0.162
WMH ~						
Activation (a3)	-0.068	0.048	-1.433	0.152	-0.068	-0.070
EffCnn ~						
Activation (a4)	-0.030	0.012	-2.491	0.013	-0.030	-0.133
Cognition ~						
HC_vol (b1)	-0.168	0.053	-3.171	0.002	-0.168	-0.175
EC_vol (b2)	-0.106	0.051	-2.083	0.037	-0.106	-0.110
WMH (b3)	-0.092	0.044	-2.084	0.037	-0.092	-0.091
EffCnn (b4)	-0.787	0.226	-3.479	0.001	-0.787	-0.177

Covariances:

	Est.	Std.Err	z-value	P(>|z|)	Std.lv	Std.all
.HC_vol ~~						
.EC_vol	0.495	0.061	8.058	0.000	0.495	0.510

Variances:

	Est.	Std.Err	z-value	P(>|z|)	Std.lv	Std.all
.Cognition	0.752	0.054	14.018	0.000	0.752	0.815
.HC_vol	0.970	0.079	12.283	0.000	0.970	0.972
.EC_vol	0.971	0.076	12.741	0.000	0.971	0.974
.WMH	0.890	0.060	14.774	0.000	0.890	0.995
.EffCnn	0.046	0.003	13.693	0.000	0.046	0.982

Defined Parameters:

	Est.	Std.Err	z-value	P(>|z|)	Std.lv	Std.all
indirect_HC	0.029	0.014	2.102	0.036	0.029	0.029
indirect_EC	0.018	0.011	1.580	0.114	0.018	0.018
indirect_WMH	0.006	0.006	1.023	0.306	0.006	0.006
indirect_EffCnn	0.023	0.011	2.057	0.040	0.023	0.024
total_indirect	0.076	0.025	3.048	0.002	0.076	0.077
direct	0.214	0.052	4.117	0.000	0.214	0.216
total	0.290	0.052	5.567	0.000	0.290	0.292

Output for the parallel mediation analysis using data from n = 407 individuals with complete cognitive, connectivity, WMH, volume and fMRI data at baseline for disease-related activation. Parameters were tested using the two-sided Wald z-statistic. Please note that p-values are uncorrected. Abbreviations: EC_vol – Entorhinal Cortex Volume; EffCnn – Effective Connectivity; HC_vol – Hippocampal Volume; indirect_HC – Indirect path via Hippocampal Volume; indirect_EC – Indirect Path via Entorhinal Cortex Volume; indirect_WMH – Indirect Path via White Matter Hyperintensities; indirect_WMH – Indirect Path via Effective Connectivity; WMH – White Matter Hyperintensities

Supplementary Table 7 | Residual variability and disease-related activity predict cognitive performance in activation regions

	Cognitive Performance (A-prime)		
Predictors	*Estimates*	*CI*	*p*
(Intercept)	0.80	0.68 – 0.91	3.32×10^{-90}
Resid. Var. (Act.)	-0.01	-0.02 – -0.00	0.004
Activation	0.15	0.11 – 0.19	2.91×10^{-12}
Age (baseline)	-0.00	-0.00 – -0.00	0.002
Sex [m]	-0.02	-0.03 – 0.00	0.056
Education (years)	0.01	0.00 – 0.01	6.90×10^{-6}
Observations	434		
R^2 / R^2 adjusted	0.224 / 0.215		

Output from the multiple linear regression using n = 434 individuals assessing the association between cognitive performance in the fMRI task and residual variability and contrast values in activation regions accounting for age, sex, and years of education. Parameters were tested using a two-sided one-sample t-test. Please note that p-values are uncorrected. Abbreviations: Resid. Var. (Act.) – Residual Variability in activation regions, CI: 95% Confidence interval.

Supplementary Table 8 | Residual variability and disease-related activity predict cognitive performance in deactivation regions

	Cognitive Performance (A-prime)		
Predictors	*Estimates*	*CI*	*p*
(Intercept)	0.89	0.77 – 1.00	4.46×10^{-91}
Resid. Var. (Deact.)	-0.01	-0.02 – -0.00	0.008
Deactivation	-0.15	-0.21 – -0.10	2.95×10^{-7}
Age (baseline)	-0.00	-0.00 – -0.00	2.22×10^{-5}
Sex [m]	-0.02	-0.04 – -0.00	0.017
Education (years)	0.01	0.00 – 0.01	1.68×10^{-4}
Observations	434		
R^2 / R^2 adjusted	0.186 / 0.176		

Output from the multiple linear regression using n = 434 individuals assessing the association between cognitive performance in the fMRI task and residual variability and contrast values in deactivation regions accounting for age, sex, and years of education. Parameters were tested using a two-sided one-sample t-test. Please note that p-values are uncorrected. Abbreviations: Resid. Var. (Deact.) – Residual Variability in deactivation regions.

Supplementary Table 9 | Linear mixed effects model output for the association between successful memory encoding-related deactivation and disease stage

	**Successful memory encoding - Deactivation**			
Predictors	*Estimates*	*CI*	*Statistic*	*p*
(Intercept)	-0.08	-0.10 – -0.05	-6.58	5.59×10^{-10}
Age (years, centered)	0.00	0.00 – 0.01	2.09	3.789×10^{-2}
Sex [m]	-0.02	-0.05 – 0.01	-1.23	0.219
Education (years, centered)	-0.00	-0.01 – 0.00	-1.43	0.155
Disease Stage	0.01	0.00 – 0.01	4.61	7.35×10^{-6}
Time	0.03	0.01 – 0.05	3.26	1.23×10^{-3}
Disease Stage × Time	0.00	0.00 – 0.01	2.72	6.93×10^{-3}
Age (years, centered) × Time	0.00	-0.00 – 0.00	1.14	0.254
Sex [m] × Time	-0.03	-0.06 – -0.01	-2.80	5.44×10^{-3}
Education (years, centered) × Time	0.00	-0.00 – 0.01	1.62	0.105
**Random Effects**				
σ^2	0.02			
$\tau_{00\ IDs}$	0.00			
ICC	0.20			
N $_{IDs}$	168			
Observations	493			
Marginal R^2 / Conditional R^2	0.104 / 0.284			

Output for the linear mixed effects model for the association between successful memory encoding – related deactivation and disease stage in a subsample of n = 168 individuals with available longitudinal fMRI data and biomarker data. Effect of interest was the interaction between Disease stage and time on longitudinal change in successful memory encoding – related deactivation. Parameters were estimated using restricted maximum likelihood. To determine whether they differ significantly from zero, two-sided t-tests were used. Here, we report the raw *p*-values.

Supplementary Table 10 | Linear mixed effects model output for the association between successful memory encoding-related activation and disease stage

	**Successful memory encoding - Activation**			
Predictors	*Estimates*	*CI*	*Statistic*	*p*
(Intercept)	0.18	0.15 – 0.22	10.08	4.188×10^{-3}
Age (years, centered)	-0.01	-0.01 – -0.00	-3.65	3.47×10^{-4}
Sex [m]	-0.00	-0.05 – 0.05	-0.00	0.997
Education (years, centered)	0.02	0.01 – 0.03	3.78	2.24×10^{-4}
Disease Stage	-0.01	-0.01 – -0.00	-3.63	3.65×10^{-4}
Time	0.00	-0.02 – 0.03	0.09	0.931
Disease Stage × Time	0.00	-0.00 – 0.01	1.47	0.141
Age (years, centered) × Time	0.00	-0.00 – 0.00	0.44	0.657
Sex [m] × Time	0.01	-0.03 – 0.04	0.39	0.697
Education (years, centered) × Time	0.00	-0.01 – 0.01	0.05	0.957
**Random Effects**				
σ^2	0.03			
$\tau_{00\ IDs}$	0.01			
ICC	0.25			
N_{IDs}	168			
Observations	490			
Marginal R^2 / Conditional R^2	0.121 / 0.341			

Output for the linear mixed effects model for the association between successful memory encoding – related activation and disease stage in a subsample of n = 168 individuals with available longitudinal fMRI data and biomarker data. Effect of interest was the interaction between Disease stage and time on longitudinal change in successful memory encoding – related activation. Parameters were estimated using restricted maximum likelihood. To determine whether they differ significantly from zero, two-sided t-tests were used. Here, we report the raw p-values.

Supplementary Table 11 | R packages used in this study

Package name	Version number
lme4	v1.1-35.3
lmertest	v3.1-3
npreg	v1.0-9
stats	v4.3.1
pracma	v2.4.4
effectsize	v0.8.6
lavaan	v0.6-19
ppcor	v1.1
emmeans	v1.8.8
dplyr	v1.1.3
ggplot2	v3.5.0
sjPlot	v2.8.15
tidySEM	v0.2.8

Supplementary Figure 1 | Model-based Gaussian-Process realizations from the model posterior distribution

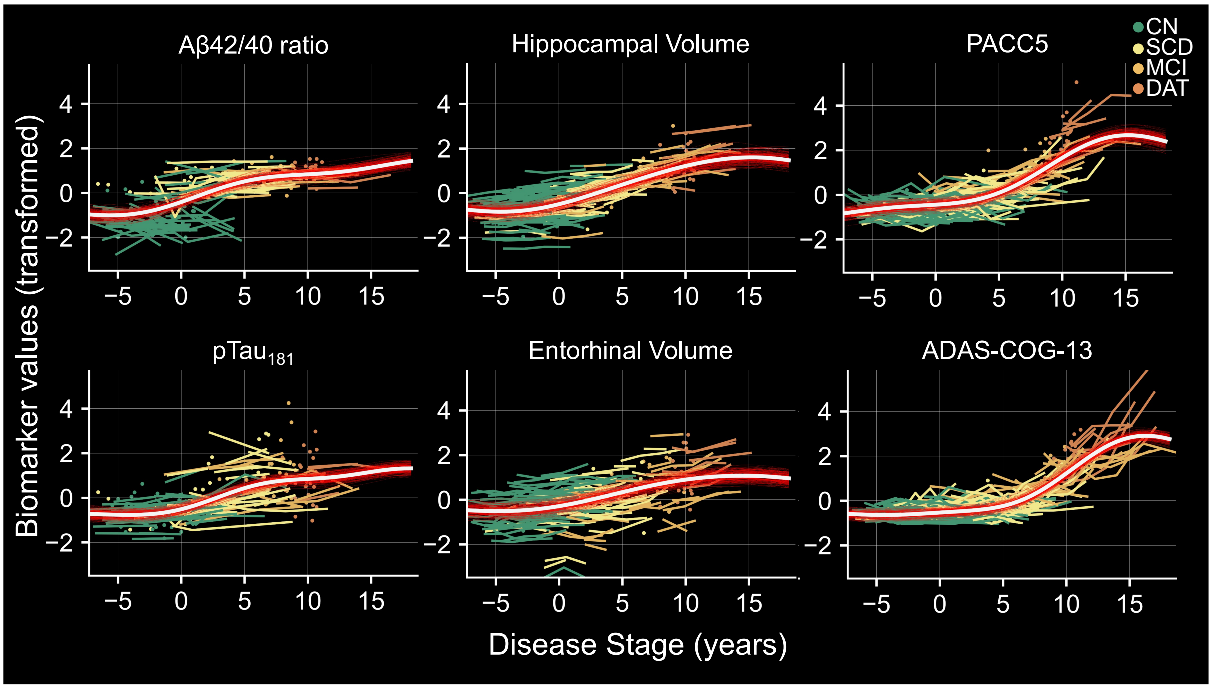

Red curves show 200 individually sampled realizations from the model posterior. Curves in white show the average GP. Colored lines indicate 739 longitudinal data points from the 208 participants added into the model. All available longitudinal data was used. CN = healthy controls, SCD = subjective cognitive decline, MCI = mild cognitive impairment, DAT = mild dementia of the Alzheimer's disease type.

Supplementary Figure 2 | Interpretability of latent disease stage

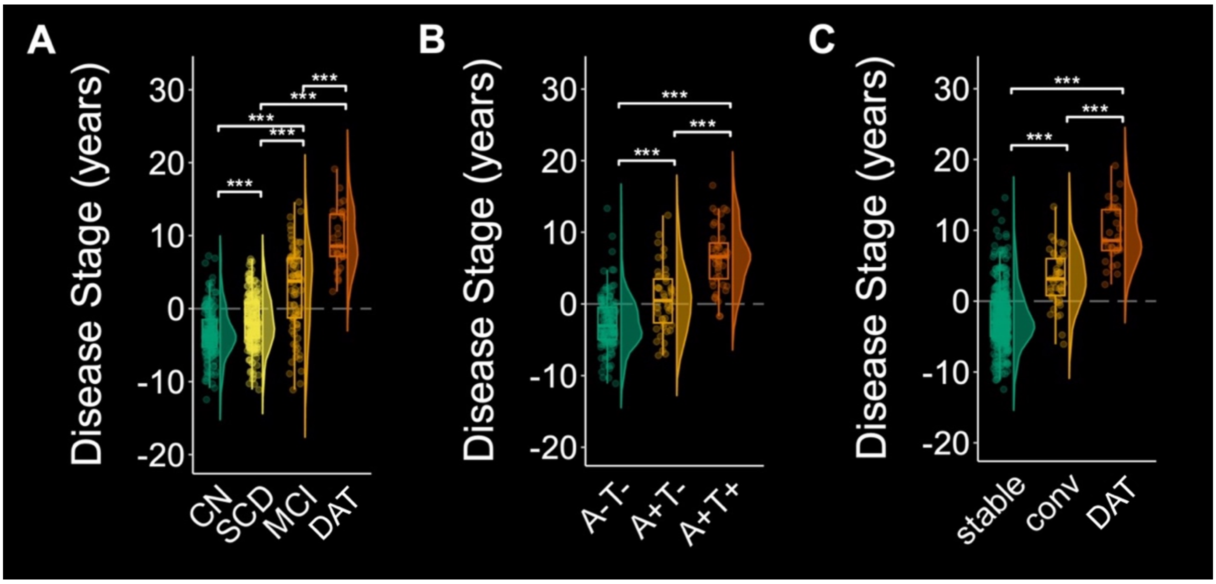

Here, we report baseline associations between clinical variables and the latent disease stage obtained from the DPM approach. On average, disease stage values indicate a change from clinically healthy to disease-related groups around the disease stage 0. **A** Association between disease groups and disease stages (N = 493). Clinically impaired participants (MCI & DAT) show larger disease stage values than clinically unimpaired participants (CN & SCD). **B** Association between AT biomarker groups and disease stage (N = 222). Equally, participants with amyloid positivity show larger disease stage values than amyloid negative participants. **C** In a subsample of N = 459 with available conversion data (n_{stable} = 358; n_{conv} = 45, n_{DAT} = 32), participants who convert to MCI or DAT show larger disease stage values than stable participants. $p < .001$***, $p < .01$**, $p < .05$*, $p > .05^{n.s.}$. Source data are provided as a source data file.

Supplementary Figure 3 | Bivariate correlations between variables used in the DPM and the DPM-derived disease stage for the model fitting sample (n = 208).

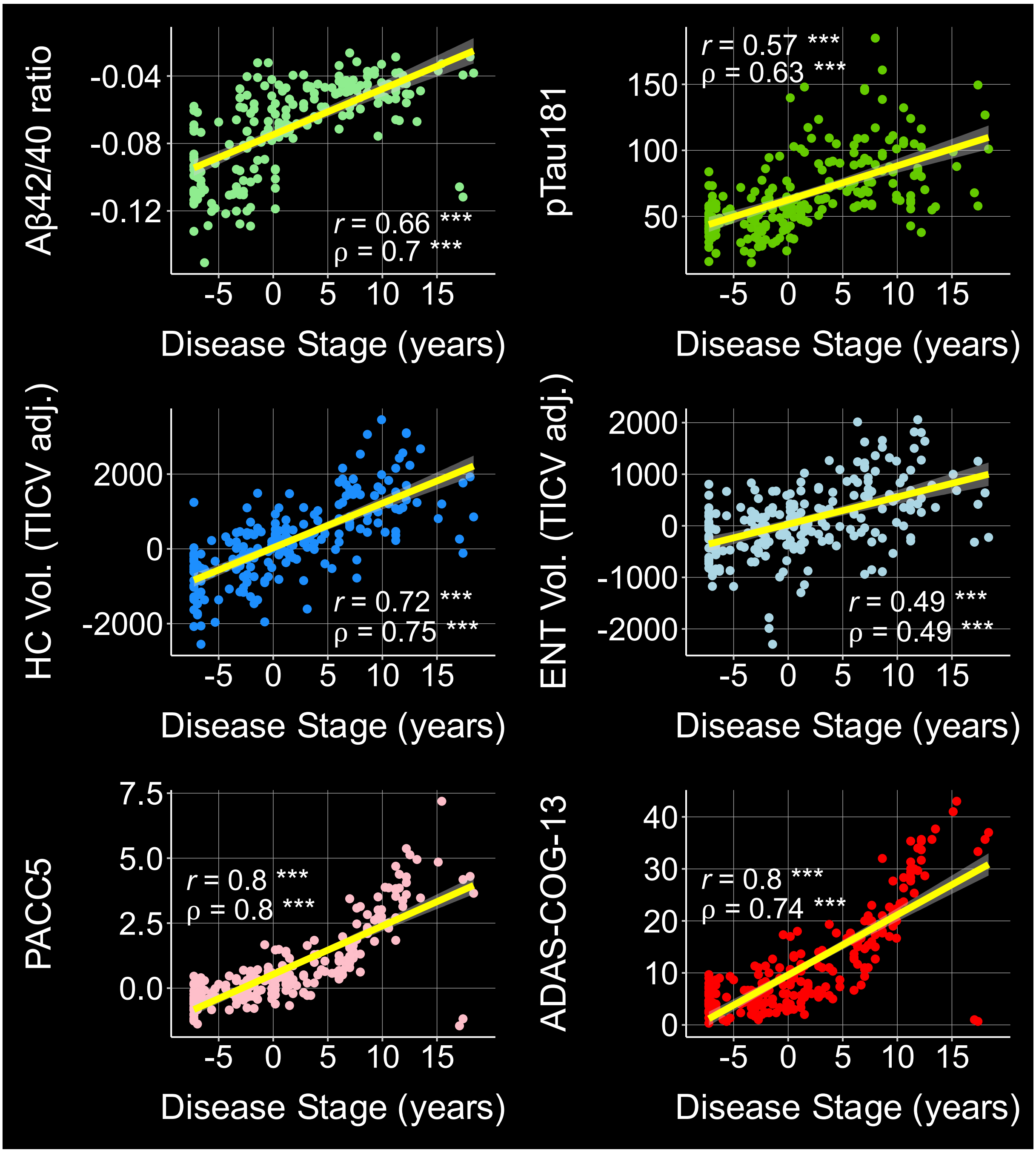

Here, we report Pearson (r) and Spearman rank (ρ) correlations for each variable in the fitting sample comprising $n_{CN} = 82$, $n_{SCD} = 57$, $n_{MCI} = 44$, $n_{DAT} = 27$ participants. Note that the CSF ratio, MTL volumes and PACC5 were multiplied with -1. Therefore, an increase in disease stage is related to an increase in more pathological DPM marker values. HC Vol.: Hippocampal Volume, ENT Vol.: Entorhinal Cortex Volume. Both volumes were adjusted for total intracranial volume (TICV adj.). $p < .001$***, $p < .01$**, $p < .05$*, $p > .05^{n.s.}$. Source data are provided as a source data file.

Supplementary Figure 4 | Non-linear associations between activation and disease stage

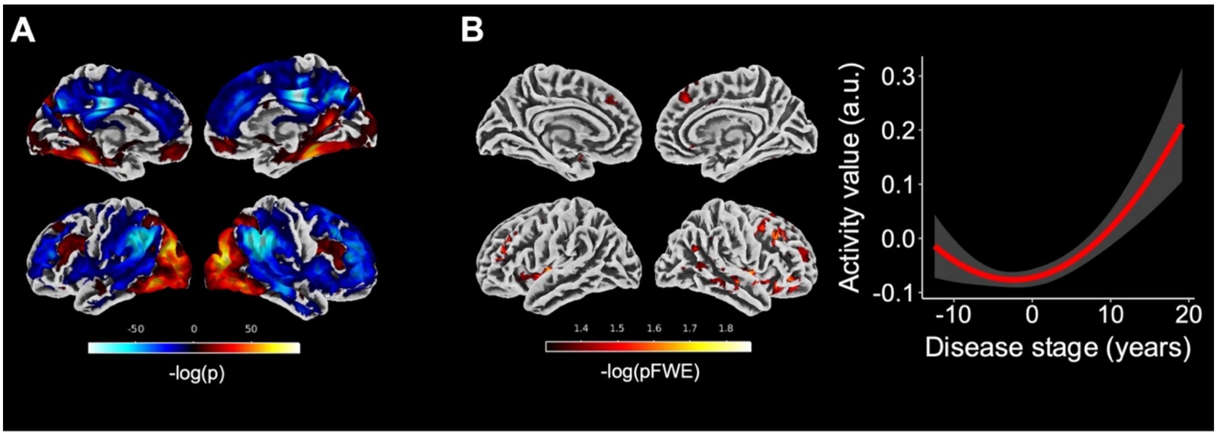

Above and beyond the reported effects, we also found non-linear u-shaped associations between deactivation and disease stages. **A** For reference, we reprinted the average activation and deactivation regions from Figure 2A. **B** U-shaped association between deactivation regions and disease stages corrected for age, sex, and years of education using all scans from n = 493 participants. Results were obtained from the main SwE model. Again, results were obtained using Wild bootstrapping with 5000 repetitions with a threshold at $p_{FWE} < 0.05$. Areas include temporal, insular and bilateral frontal cortices (left). Values are depicted as mean ± SEM. Source data are provided as a source data file.

Supplementary Figure 5 | Towards biomarker progression curves for activation independent of age

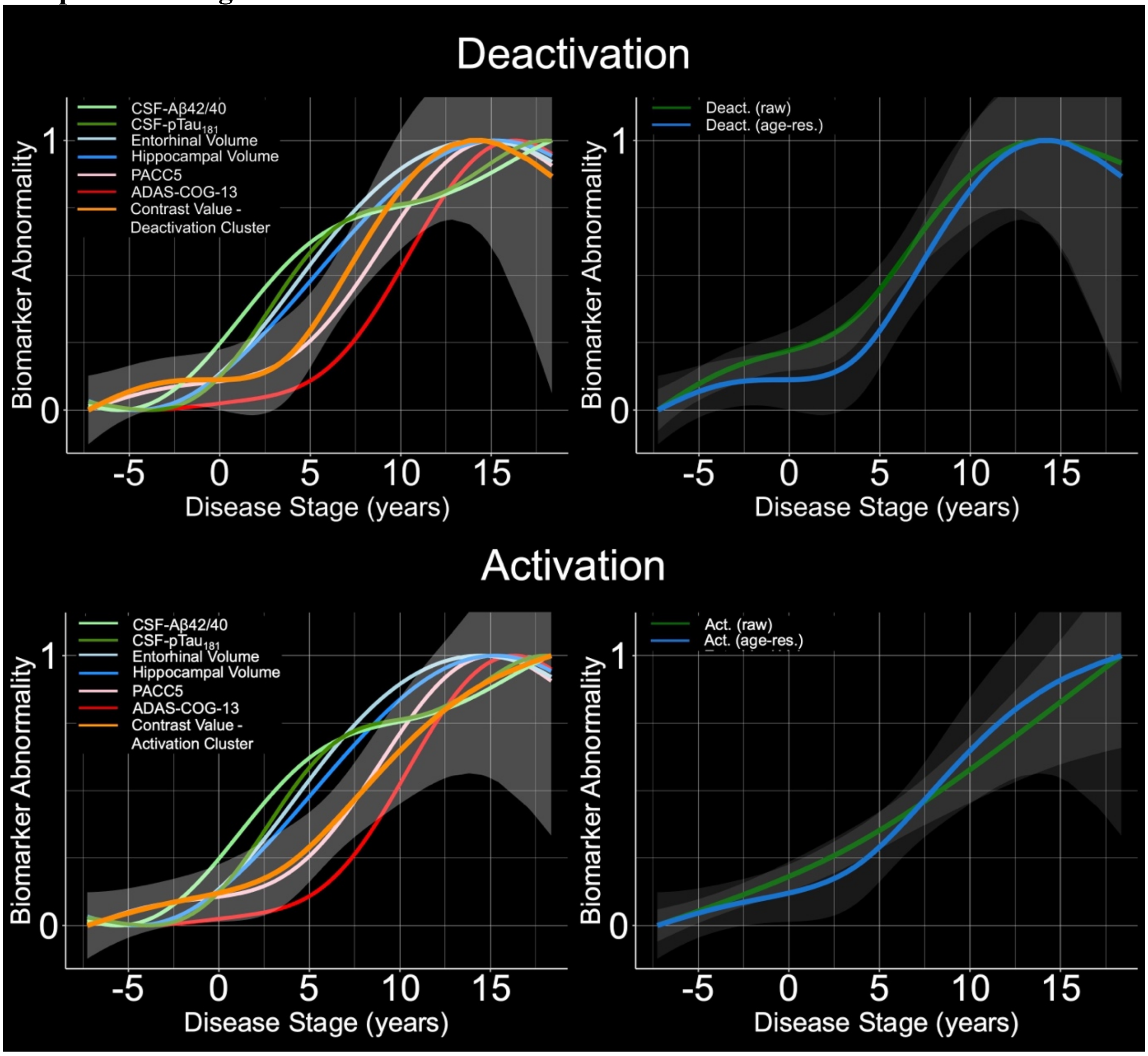

Age-independent activity curves. The curves were generated using smoothing splines with adjustments for age. In general, it can be seen that cognition is preceded by activation, tau pathlogy, volume, and, at last, amyloid pathology. When correcting for age the abnormality curve for deactivation shows an attenuated initial increase in abnormality in the earliest of disease stages (top, blue) in comparison to the curve including age-related variance. Results were obtained by using smoothing splines from the npreg R package (n = 493). For activation, the association over the disease stages did not change. Source data are provided as a source data file.

Supplementary Figure 6 | Parallel mediation analyses reveal volume and connectivity contributions to the association between activity and AD progression scores

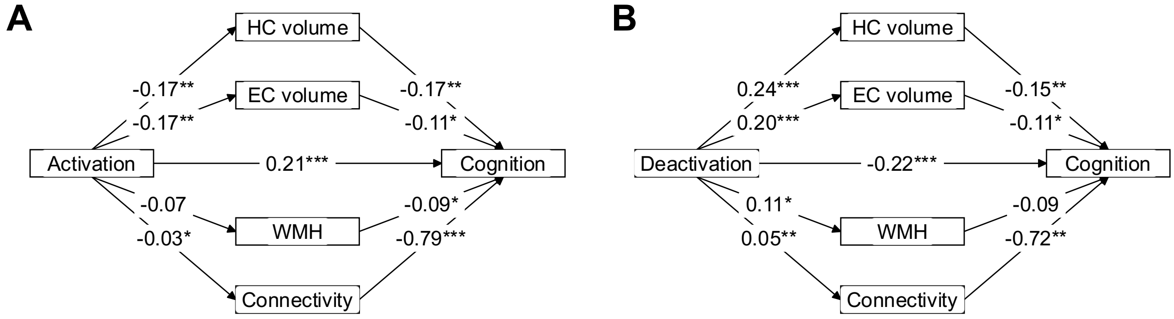

Using a subsample of 407 individuals with complete activation, connectivity, volumetry and cerebrovascular data, we calculated parallel mediations for the association between activity and cognitive performance. WMH values were log10 transformed prior to model fit. Additionally, all variables were residualized for age, sex, and education and standardized before entering into the model. Standard errors were estimated using bootstrapping with 5000 repetitions. **A** Parallel mediation model for the association between activation and cognitive performance reveals that hippocampal volume and connectivity, but not entorhinal volume or WMH partially mediate the association. **B** For deactivation, a similar partial mediation structure could be observed. Connectivity and hippocampal volume partially mediated the association between deactivation and cognitive performance, but not WMH or entorhinal volume. P-values can be found in Supplementary Tables 5 and 6. Source data are provided as a source data file.

Supplementary Figure 7 | Schematic representation of analyses in our study

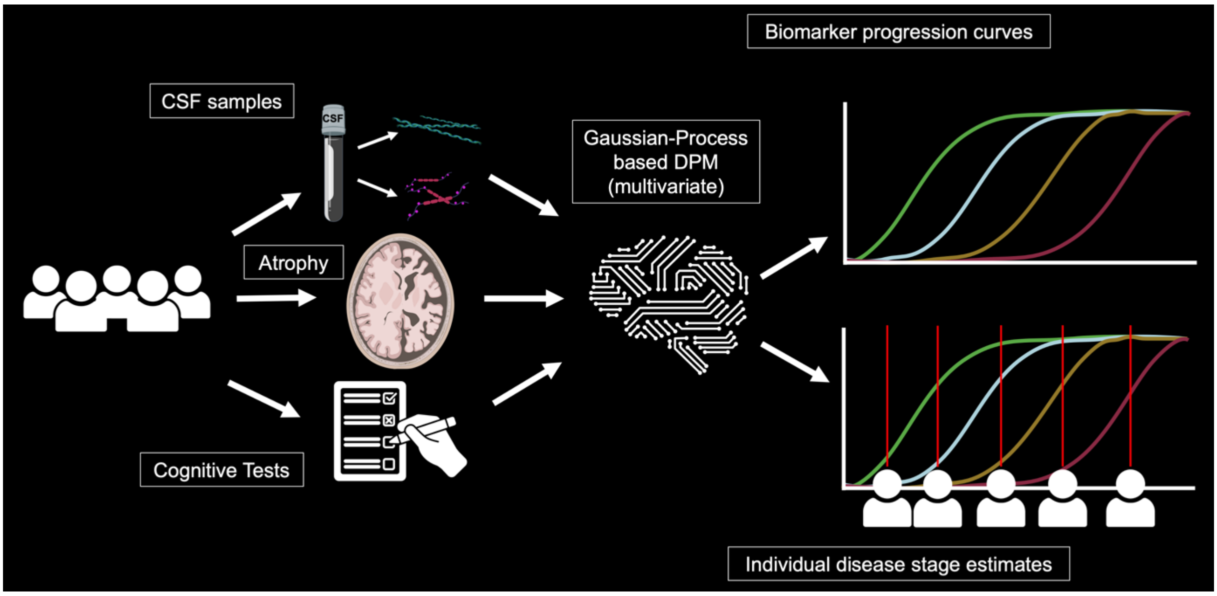

For model fit, we selected 208 participants with at least one complete measurement occasion of biomarkers (see Lorenzi et al., 2019). If available, we also included longitudinal biomarker information, such that 739 data points were included in the model training procedure. Model training parameters were kept at default values: Outer iterations = 6; Inner iterations = 200; trade-off = 100. The trade-off describes the strength of the monotonicity constraint in relation to the model fit. Monotonicity constraint for each biomarker was set to 1. This was done such that increasing levels in biomarkers would be associated with higher burdens in all respective domains. For this, the CSF-Aβ42/40 ratio and volume values were multiplied by -1. After model fitting, the disease stage estimates for all participants were obtained using the *Predict* method. Lastly, time was operationalized as time-since-baseline expressed in years. All variables were z-standardized prior to model fitting. Created in BioRender. Lattmann, R. (2026) https://BioRender.com/p996rzj
