# Supplementary material for: Dysfunction of the episodic memory network in the Alzheimer’s disease cascade
Source: Nat Commun. 2026 Apr 17;17:3578. doi: 10.1038/s41467-026-71831-z (PMC13090369; doi:10.1038/s41467-026-71831-z)
Supplement: Supplementary file 2 — Reporting Summary [file 41467_2026_71831_MOESM2_ESM.pdf]

nature portfolio

Corresponding author(s): René Lattmann

Last updated by author(s): Feb 24, 2026

Reporting Summary

Nature Portfolio wishes to improve the reproducibility of the work that we publish. This form provides structure for consistency and transparency in reporting. For further information on Nature Portfolio policies, see our Editorial Policies and the Editorial Policy Checklist.

Statistics

For all statistical analyses, confirm that the following items are present in the figure legend, table legend, main text, or Methods section.

n/a	Confirmed	
☐	☒	The exact sample size (*n*) for each experimental group/condition, given as a discrete number and unit of measurement
☐	☒	A statement on whether measurements were taken from distinct samples or whether the same sample was measured repeatedly
☐	☒	The statistical test(s) used AND whether they are one- or two-sided<br>*Only common tests should be described solely by name; describe more complex techniques in the Methods section.*
☐	☒	A description of all covariates tested
☐	☒	A description of any assumptions or corrections, such as tests of normality and adjustment for multiple comparisons
☐	☒	A full description of the statistical parameters including central tendency (e.g. means) or other basic estimates (e.g. regression coefficient) AND variation (e.g. standard deviation) or associated estimates of uncertainty (e.g. confidence intervals)
☐	☒	For null hypothesis testing, the test statistic (e.g. *F*, *t*, *r*) with confidence intervals, effect sizes, degrees of freedom and *P* value noted<br>*Give P values as exact values whenever suitable.*
☒	☐	For Bayesian analysis, information on the choice of priors and Markov chain Monte Carlo settings
☒	☐	For hierarchical and complex designs, identification of the appropriate level for tests and full reporting of outcomes
☐	☒	Estimates of effect sizes (e.g. Cohen's *d*, Pearson's *r*), indicating how they were calculated

Our web collection on statistics for biologists contains articles on many of the points above.

Software and code

Policy information about availability of computer code

Data collection	For stimuli presentation and for response collection in the fMRI task, "Presentation" (Neurobehavioral Systems Inc.) was used. Depending on the acquisition sites, different versions were used (14.2, 14.9, 16.2, 16.3, 16.5, 17.1, 18.1)
Data analysis	We used SPM12 and its extension of the sandwich estimator toolbox (v.2.2.2) and MatlabR2022b for fMRI data preprocessing and analysis. To generate the disease progression model, we used custom code from Lorenzi et al. (2021) available from https://gitlab.inria.fr/epione/GP_progression_model_V2 in Python version 3.9. R version 4.2.1 was used for statistical analyses (packages lme4 v1.1-35.3, lmertest v3.1-3, npreg v1.0-9, stats v4.3.1, pracma v2.4.4, effectsize v0.8.6, lavaan v0.6-19, ppcor v1.1, emmeans v1.8.8), data organization (dplyr v1.1.3) and visualization (ggplot2 v3.5.0, sjPlot v2.8.15, tidySEM v0.2.8).

For manuscripts utilizing custom algorithms or software that are central to the research but not yet described in published literature, software must be made available to editors and reviewers. We strongly encourage code deposition in a community repository (e.g. GitHub). See the Nature Portfolio guidelines for submitting code & software for further information.

Data

Policy information about availability of data

All manuscripts must include a data availability statement. This statement should provide the following information, where applicable:

- Accession codes, unique identifiers, or web links for publicly available datasets
- A description of any restrictions on data availability
- For clinical datasets or third party data, please ensure that the statement adheres to our policy

The raw data collected in the study "DELCODE—DZNE-Longitudinal Cognitive Impairment and Dementia Study (BN012)" cannot be made openly available without violation of the data protection concept of the DZNE. The same applies to the processed individual (f)MRI images. Access to the relevant study data can be obtained by submitting an application to the Clinical Research Platform of the DZNE. The template for the application for the submission of data and biomaterial samples is available on the DZNE homepage (https://www.dzne.de/en/research/research-areas/clinical-research/databases-of-the-clinical-research/). The expected timeframe for response to access requests is 1 month. Access will be granted for 10 years. Source data are provided with this paper.

Research involving human participants, their data, or biological material

Policy information about studies with human participants or human data. See also policy information about sex, gender (identity/presentation), and sexual orientation and race, ethnicity and racism.

Reporting on sex and gender	Gender was not collected. Sex was self-reported with the distribution of 257 femals and 236 males in our analysis sample. We did not perform sex-specific analyses, as the goal of our study pertained to a broader understanding of episodic memory circuit dysfuntion in the AD cascade in general.
Reporting on race, ethnicity, or other socially relevant groupings	Race or ethnicity were not collected in the data collection process. We did not use any other socially relevant information other than years of education. Together with age and sex, these demographics were controlled for in our analysis.
Population characteristics	See below
Recruitment	All patient groups (SCD, MCI, AD) were referrals, including self-referrals, to the participating memory centers. Standardized public advertisement was used for healthy control participants and relatives of AD dementia patients.
Ethics oversight	The process was led and coordinated by the ethical committee of the medical faculty of the University of Bonn (trial registration number 117/13).

Note that full information on the approval of the study protocol must also be provided in the manuscript.

Field-specific reporting

Please select the one below that is the best fit for your research. If you are not sure, read the appropriate sections before making your selection.

☐ Life sciences ☒ Behavioural & social sciences ☐ Ecological, evolutionary & environmental sciences

For a reference copy of the document with all sections, see nature.com/documents/nr-reporting-summary-flat.pdf

Behavioural & social sciences study design

All studies must disclose on these points even when the disclosure is negative.

Study description	The DZNE Longitudinal Cognitive Impairment and Dementia study (DELCODE; Jessen et al., 2018) is an observational longitudinal memory clinic-based multicenter study in Germany. The analysed data from the study is quantitative.
Research sample	N = 493 participants from the DELCODE study that come from the general German population over 60 years. As the DELCODE study was particularly designed at investigating participants with subjective cognitive decline, these are over-represented in the sample. The sample is well educated with 14.7 years of education (SD: 2.9 years). 52.1% of the participants were female and on average 70.64 years old (SD: 5.6 years). We utilized this study because of its suitability, i.e. availability of annual task-fMRI data of a visual memory encoding task, co-availability of longitudinal CSF-based biomarkers and longitudinal structural volume data allowing for a multimodal representation of AD across the broad AD-risk spectrum from healthy controls to AD dementia.
Sampling strategy	Stratified sampling was used to enroll 1011 participants into 5 groups at baseline: subjective cognitive decline (SCD), mild cognitive impairment (MCI), dementia due to Alzheimer's disease (AD), AD patient relatives and cognitively normal controls. The sample size was determined to be powered for the univariate detection of significant predictors of cognitive decline in subjects with SCD. In one multicenter memory clinic study in Europe the frequency of AD type CSF in subjects with SCD was 50%. Due to slight differences in the definition of SCD (i.e. inclusion of subjects reporting worries about other that memory decline in DELCODE) a frequency of 40% individuals in the SCD group that will display evidence for amyloid deposition in the CSF was estimated. One study reported a hazard ratio (HR) of 15 for MCI/dementia (evidenced by episodic memory decline) in memory clinic patients with SCD and Aß42 reduction in the CSF with a mean observation period of 4 years (van Harten et al., 2011). In the present multicenter study, the estimation was more conservative. The assumption for an univariate predictor for episodic memory decline in preclinical AD over 5 years was an odds ratio of 3. With these assumptions (40% SCD subjects with preclinical AD, OR=3, 5 year follow-up, 10% drop-out) 300 patients

	with SCD are required to identify a predictor of episodic memory decline with 80% power. The sizes of the other groups were defined to be sufficiently large for comparison with the SCD group and to be feasible to recruit within the DZNE multicenter structure.
Data collection	A trained researcher administered the neuropsychological tests (pen and paper). The researchers were not aware of the primary study hypothesis. A researcher recorded the participants' responses in the post-fMRI retrieval task on a computer. The researcher merely recorded the responses and was unaware of the images the subject had seen in the scanner. No one was present except for the researcher and the participant.
Timing	Data collection started in 2014 and ended in 2023
Data exclusions	N = 23 participants with a CSF biomarker profile outside the Alzheimer's continuum (see Jack et al., 2018) were excluded. For the estimation of the disease progression model, we followed the rationale by Lorenzi et al. (2019) by including only participants who have at least one full measurement occasions of CSF, volumetric, and cognitive performance data. Additionally, clinical conversion data was used to exclude participants who later converted to non-amnestic MCI (n = 12) or to non-Alzheimer's type dementia (n = 7): Dementia with Lewy Bodies (n = 1), Frontotemporal dementia (n=1), "unknown" dementia (n = 5). Finally, we restricted clinical AD-risk groups (i.e. SCD, MCI, Alzheimer's disease) to participants who were amyloid positive.<br>For our final analysis sample, we did not include participants who were relatives of AD patients (n = 80), because we did not have a-priori hypotheses regarding this subsample. The overall analysis sample was restricted to those with available fMRI data.
Non-participation	Decline of participation has not been recorded. 210 participants dropped out of the study for various reasons (119: participant or relative wanted termination; 19: participant is in nursing home; 16: other disease that prevents further participation; 11: decision of the responsible doctor; 7: participant moved away; 6: contact lost; 3: participant is bedridden; 29: other reasons, e.g. death)
Randomization	Participants were not allocated to experimental groups.

Reporting for specific materials, systems and methods

We require information from authors about some types of materials, experimental systems and methods used in many studies. Here, indicate whether each material, system or method listed is relevant to your study. If you are not sure if a list item applies to your research, read the appropriate section before selecting a response.

Materials & experimental systems

n/a	Involved in the study	
☒	☐	Antibodies
☒	☐	Eukaryotic cell lines
☒	☐	Palaeontology and archaeology
☒	☐	Animals and other organisms
☒	☐	Clinical data
☒	☐	Dual use research of concern
☒	☐	Plants

Methods

n/a	Involved in the study	
☒	☐	ChIP-seq
☒	☐	Flow cytometry
☐	☒	MRI-based neuroimaging

Plants

Seed stocks	*Report on the source of all seed stocks or other plant material used. If applicable, state the seed stock centre and catalogue number. If plant specimens were collected from the field, describe the collection location, date and sampling procedures.*
Novel plant genotypes	*Describe the methods by which all novel plant genotypes were produced. This includes those generated by transgenic approaches, gene editing, chemical/radiation-based mutagenesis and hybridization. For transgenic lines, describe the transformation method, the number of independent lines analyzed and the generation upon which experiments were performed. For gene-edited lines, describe the editor used, the endogenous sequence targeted for editing, the targeting guide RNA sequence (if applicable) and how the editor was applied.*
Authentication	*Describe any authentication procedures for each seed stock used or novel genotype generated. Describe any experiments used to assess the effect of a mutation and, where applicable, how potential secondary effects (e.g. second site T-DNA insertions, mosiacism, off-target gene editing) were examined.*

Magnetic resonance imaging

Experimental design

Design type	Task-based fMRI, event-related design
Design specifications	132 trials (stimuli) per subject in one session. Each stimulus was presented for 2500ms and the inter-stimulus-interval was jittered around an average of 1250ms with a standard deviation of about 700ms.
Behavioral performance measures	Button press and response time were registered during the task. More than 8 errors in their indoor/outdoor judgement were interpreted as a lack of attention or confusion. Thus, these individuals with extreme outliers in the distribution of

indoor/outdoor errors were excluded from the analyses.

Acquisition

Imaging type(s)	Structural and functional
Field strength	3.0T
Sequence & imaging parameters	T1-weighted: magnetization prepared rapid gradient echo (MPRAGE), 1mm^3 isotropic, sagittal, TR 2500ms, TE 437ms, Flip Angle 7 degrees.<br>T2-weighted: turbo spin echo, 0.5x0.5x1.5 mm^3, coronal, TR 3500ms, TE 354ms, Flip Angle 120 degrees.<br>task fMRI: gradient echo, 2D EPI, 3.5mm^3 isotropic, oblique axial/AC-PC aligned, TR 2580ms, TE 30ms, Flip Angle 80 degrees, 206 volumes.
Area of acquisition	T1-weighted and fMRI were whole-brain, the T2-weighted scan covered the medial temporal lobe region. The region was determined visually on the T1-weighted scans.

Diffusion MRI ☐ Used ☒ Not used

Preprocessing

Preprocessing software	SPM12 in MatlabR2022b
Normalization	Non-linear transformation to a study specific template aligned with MNI space
Normalization template	Study specific template affine transformed to MNI152
Noise and artifact removal	Unwarping of functional images using voxel displacement maps derived from fieldmaps to correct for image distortions.
Volume censoring	No volume censoring was performed.

Statistical modeling & inference

Model type and settings	Second-level mass univariate: We modelled second-level subsequent memory contrast images using Time, linear and quadratic predictor of disease stage, age, sex, education, and the interactions between the demographics and time.
Effect(s) tested	Effects of interest were the associations of disease stage with the subsequent memory contrast.

Specify type of analysis: ☐ Whole brain ☐ ROI-based ☒ Both

Anatomical location(s)	Regions-of-interest were selected based on the second-level analysis of the first-level subsequent memory contrast maps from in the sandwich estimator. Voxels with a beta value statistically different from 0 (Family Wise Error - corrected p < 0.05) were considered.
Statistic type for inference (See Eklund et al. 2016)	Threshold-free cluster enhancement with Wild bootstrap (1000 repetitions; Guillaume et al., 2017).
Correction	Family-Wise error (p < 0.05).

Models & analysis

n/a	Involved in the study
☐	☒ Functional and/or effective connectivity
☒	☐ Graph analysis
☐	☒ Multivariate modeling or predictive analysis

Functional and/or effective connectivity	Effective connectivity from dynamic causal modelling of a previous study (Suksangkharn et al., 2024). This study assessed effective connectivity in the DELCODE sample during successful memory encoding and its dependency on AD biomarkers. In essence, the DCM contained the nodes hippocampus, parahippocampal place area and precuneus. In this study, we used the effective connectivity from the right parahippocampal place area to the right precuneus of the A matrix of the DCM.
Multivariate modeling and predictive analysis	We obtained disease stage estimates using a pre-existing disease progression model framework based on Gaussian Processes (Lorenzi et al., 2019). Input variables within our model building sample (see above) were longitudinal CSF biomarker information (Amyloid beta 42/40 ratio, Phosphotau 181), longitudinal morphometric information (hippocampal volume, entorhinal cortex volume), and cognition data (PACC5, ADAS-COG-13 sum score). Model fit was done in an iterative approach using standard settings (6 outer iterations, inner iterations = 200, trade off between monotonicity of biomarker curves and data fit = 100).
